# Association between the distal tibiofibular syndesmosis morphology classification and ankle osteoarthritis: a retrospective study

**DOI:** 10.1186/s13018-023-03985-1

**Published:** 2023-08-03

**Authors:** Lei Huang, XiaoHong Zhang, Siyi Yang, Jiwen Qing, Wangyu Wu, Houyin Shi, Dingxuan Wang, Lei Zhang

**Affiliations:** 1 School of Physical Education, Southwest Medical University, Luzhou, 646000 Sichuan Province China; 2 School of Traditional Chinese and Western Medicine, Southwest Medical University, Luzhou, 646000 Sichuan China; 3 Department of Orthopedics, The Affiliated Traditional Chinese Medicine Hospital of Southwest Medical University, 182 Chun Hui Road, Luzhou, 646000 Sichuan Province China; 4 Center for Orthopedic Diseases Research, The Affiliated Traditional Chinese Medicine Hospital of Southwest Medical University, Luzhou, 646000 Sichuan China

**Keywords:** Distal tibiofibular syndesmosis, Ankle osteoarthritis, Morphology classification

## Abstract

**Background:**

Syndesmosis injury is proposed to contribute to ankle stability and osteoarthritis (OA). However, whether distal tibiofibular syndesmosis structure is closely related to ankle OA is unclear. We hypothesized that different DTS morphology classifications would affect the biomechanics properties in ankle OA. The study aimed to determine the association between the distal tibiofibular syndesmosis (DTS) morphological classification and ankle OA.

**Methods:**

This is a retrospective study examining imaging data of 147 patients (87 males and 60 females) with ankle OA. Magnetic resonance imaging was used to access the DTS morphological classification, according to measuring various parameters. Joint space narrowing and osteophytes were measured using ankle weight-bearing radiography. The classification and parameters were analyzed to determine the relationship between the syndesmosis classification and the abnormality of ankle OA.

**Results:**

Five morphological classifications of the DTS, including Chevron (19.6%), Widow’s peak (16.2%), Flat (22.3%), Trapezoid (32.0%), and Crescent (19.6%), were shown. There were statistical differences between DTS classification and tibial angle surface angle (TAS) (*P* = .009) and talar tilt angle (TTA) (*P* = .014). The TAS (degree) of the Crescent (86.47 ± 3.21) was less than Chevron (88.75 ± 2.72) (*P* = .006), Widow’s peak (89.26 ± 3.15) (*P* = .001), Flat (88.83 ± 3.62) (*P* = .003) and Trapezoid (88.11 ± 2.62) (*P* = .041), respectively. The TTA (degree) of Crescent (86.83 ± 5.30) was less than Chevron (89.28 ± 2.46) and Widow’s peak (89.82 ± 3.41). The men were greater than women for TAS (*P* = .008) and angle (*P* = .003), which are consistent with osteophyte (*P* = .019) and the modified Kellgren–Lawrence grades (*P* = .041) between gender.

**Conclusions:**

DTS morphological classification might affect the biomechanics properties in TAS and TTA in ankle OA. In clinical practice, surgeons should pay attention to the effects of DTS on ankle OA.

*Level of Evidence*: Level III, retrospective study.

## Introduction

Osteoarthritis (OA) of the ankle, a chronic disease, is 1% of all OA cases worldwide [1]. Ankle OA is uncommon but can lead to severe dysfunction if it progresses to an advanced stage. It usually presents subchondral cysts, cartilage and subchondral bone damage, joint space narrowing, and marginal osteophytes [2–5]. Ankle OA differs from OA of the hip or knee and is usually a result of trauma, such as ankle fractures or ligament lesions. It is generally attributed to the anatomy and weight-bearing characteristics of the ankle. The ankle complex involves the tibiofibular, distal tibiofibular syndesmosis (DTS), and subtalar joint. DTS is defined as a micro-movement joint formed by the tibiofibular and four ligaments, including anteroinferior tibiofibular ligament (AITFL), posteroinferior tibiofibular ligament (PITFL) interosseous ligament (IOL), inferior transverse ligament (ITL) [6]. They play a key in maintaining the stability of the ankle. Any one of the ligaments injured can result in syndesmosis instability [7, 8].

Besides, the distal tibiofibular joint is variable in the physiological state with the ankle joint flexion extension and rotation movement [9, 10]. Usually, the movement of the fibula at the DTS can accommodate the shape of the talus [11]. The joint will widen about 1–2 mm at the mortise [12]. The width of the ankle varies with the shape of the tibial tubercle and the peroneal notch, and the widening of the ankle joint can cause ankle instability [10]. The ankle cartilage is prone to damage due to abnormal stress, leading to the development of post-traumatic osteoarthritis (PTOA) [13]. At the same time, the alignment of lower limbs is also essential for ankle OA. Some scholars have found that the tibial lateral surface angle (TLS) is greater or smaller will lead to subluxation or anterior dislocation of the talus and change the stress distribution of the ankle, thus leading to ankle OA [14]. And another study showed that the greater depth of the fibular notch might be a risk factor for ankle instability [15]. Therefore, we wondered whether different morphological features of the DTS were associated with changes in ankle joint structure, more likely leading to ankle OA.

Five DTS classifications, including Flat, Crescent, Trapezoid, Widow’s Peak, and Chevron [16]. However, the biomechanics of joint classifications has not been reported, and their role in ankle OA especially PTOA remains unclear. We hypothesized that different DTS morphology classifications would affect the biomechanics properties in ankle OA. The study aimed to confirm the association between the DTS classification and ankle OA.

## Materials and methods

### Patients

This is a retrospective study examining the imaging data of 147 patients in our institution with secondary ankle OA were included from June 2021 to March 2022 (87 males and 60 females). The average age is 48 (range, 18–60) years. The inclusion criteria included patients who met the criteria for ankle OA (mainly secondary to ankle fractures, ligament injury and chronic degeneration) by clinical and radiologic diagnosis [17]. The exclusion criteria were rheumatoid arthritis, foot and ankle infection, foot deformity, or osteochondral lesions of the talus.

### Anthropometrics

We measured the participants’ height, and the data should be accurate to 1 cm. Using a pair of electronic scales patient's weight was also precise to 1 kg (without heavy clothing).

### MRI scans

**Fig. 1:**
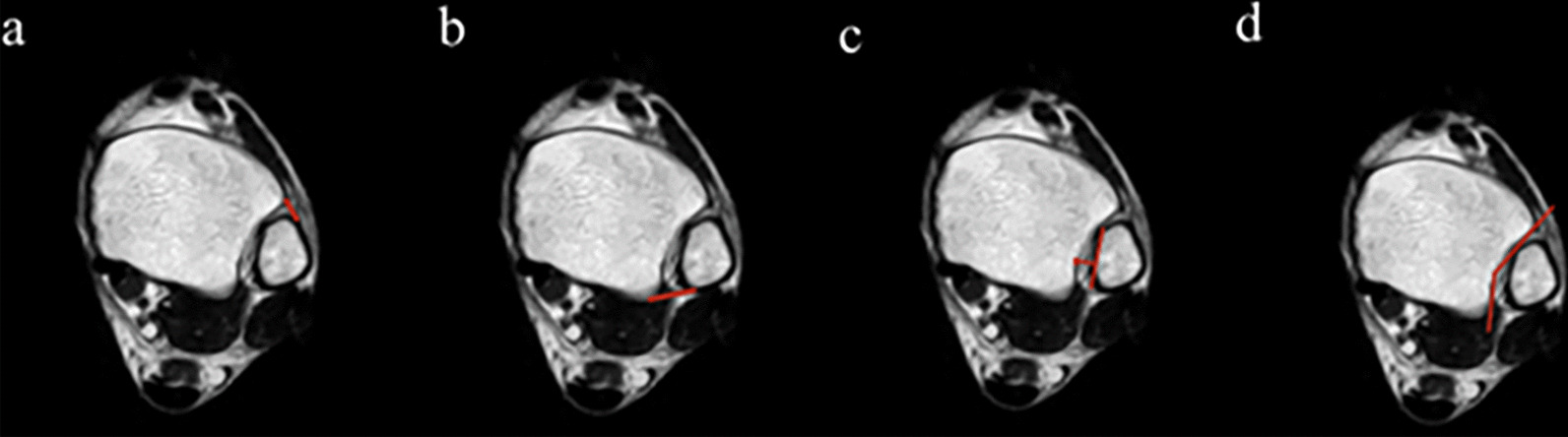
The following parameters were measured at a 1 cm proximal level to the tibial platfond. **a** Distance between anterior tibial tubercle (Chaput) and most anterior tubercle of the fibula; **b** distance between posterior tibial tubercle (Volkmann) and most posterior tubercle of the fibula; **c** maximum vertical distance between the medial fibular cortex and the anterior and posterior tubercle tips of the tibia; **d** and the angle, between the anterior and posterior facets of the fibular notch of the tibia

DTS morphological classifications were assessed through MRI scans on T1-weighted horizontal images (Philips Intra MR 1.5 T), which were classified into five classifications, including Chevron, Widow’s peak, Flat, Trapezoid, and Crescent according to Liu et al. [16]. Two investigators contemporaneously classified the DTS on account of morphological characteristics. If there were disagreements, the third person would evaluate. The classification and the following parameters were measured at 1 cm proximal level to the tibial plafond [18]: Distance between anterior tibial tubercle (Chaput) and most anterior tubercle of the fibula (a); distance between posterior tibial tubercle (Volkmann) and most posterior tubercle of the fibula (b); maximum vertical distance between the medial fibular cortex and the anterior and posterior tubercle tips of the tibia (c); and the angle, between the anterior and posterior parts of the fibular notch of the tibia (d) (Fig. 1).

### Ankle radiographic assessment

**Fig. 2:**
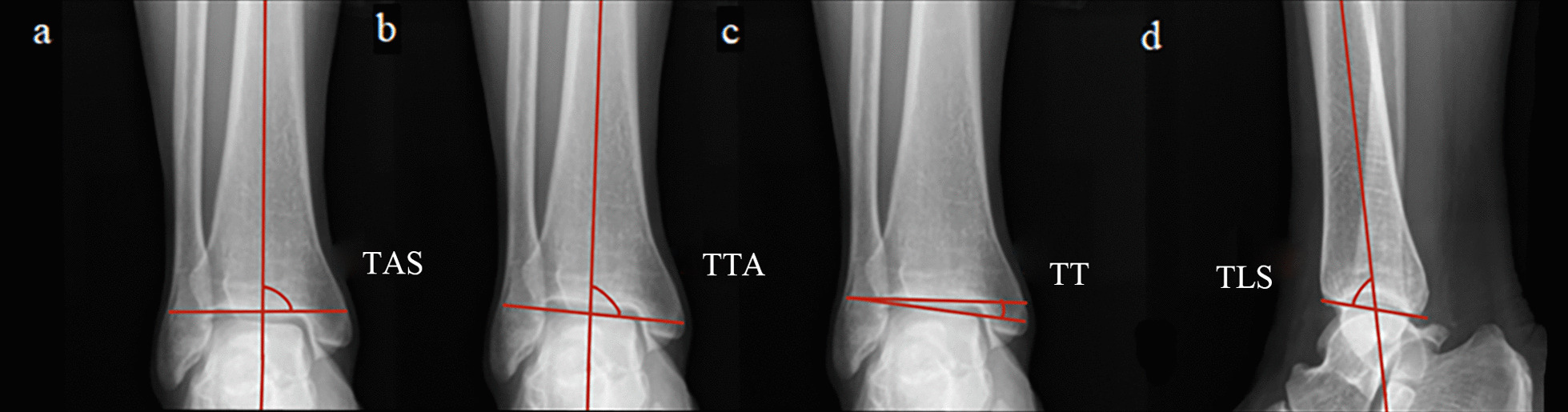
The angle is mainly measured by the ankle weight-bearing radiographs. **a** TAS: Tibial articular surface angle; **b** TTA: talar tilt angle; **c** TT: tibiotalar surface angle; **d** TLS: tibial lateral surface angle

The German Siemen Ysio DR was used to acquire radiographs. We measured for the ankle space narrowing and osteophytes with a grade of 0–3 (from normal to most serious) by the Atlas of Radiographic Features of Osteoarthritis of the Ankle and Hindfoot with Kraus et al. [19]. If there are multiple osteophytes, select the most severe one for grading. Besides, the tibial articular surface angle (TAS) (a), talar tilt angle (TTA) (b), tibiotalar surface angle (TT) (c), and tibial lateral surface angle (TLS) (d) were also measured by the anteroposterior or lateral weight-bearing view of the ankle (Fig. 2) [20–22].

### Statistical analysis

The intraclass correlation coefficient (ICC) was used to test the interobserver reliability. For the explanation of ICC value, it was poor for values < 0.40, fair for values between 0.40 and 0.59, good for values between 0.60 and 0.74, and excellent for values between 0.75 and 1.0 [23]. The normal distribution was assessed by the Shapiro-Wilks test. Data following a normal distribution will be shown as the mean ± standard deviation (SD). The continuous variable was analyzed by one-way ANOVA or student’s test. The chi-square test examined the categorical variable. It was considered a significant difference when P < 0.05. All data were evaluated by SPSS 23.0 software.

## Results

**Fig. 3:**
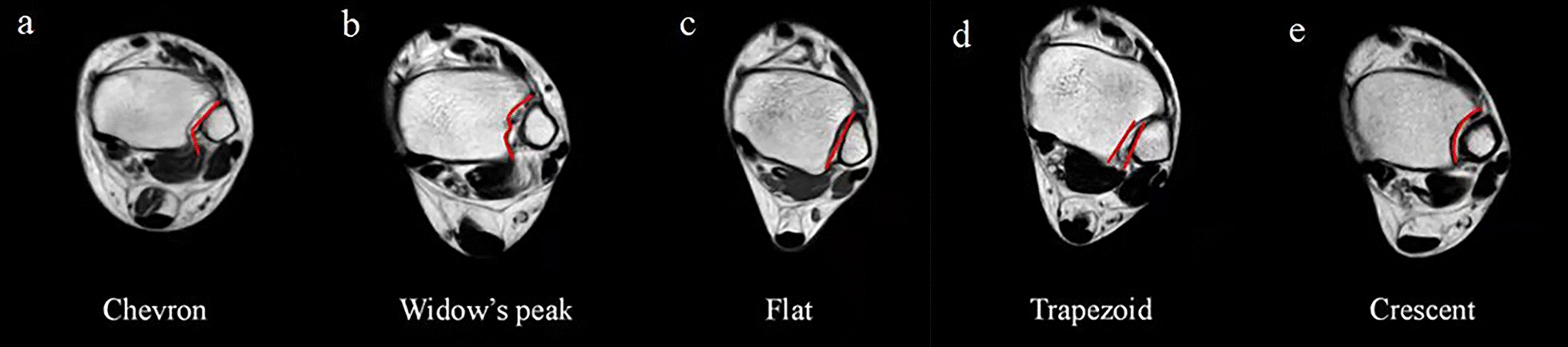
Five DTS morphological classifications. **a** Chevron; **b** Widow’s peak; **c** Flat; **d** Trapezoid; **e** Crescent

A total of 147 participants were contained, including five DTS morphology classifications, Chevron (19.7%), Widow’s peak (16.3%), Flat (22.4%), Trapezoid (21.8%), and Crescent (19.7%) were observed on MRI. The anterior and posterior tubercles of the fibula notch show a V-shape (Chevron); The fibula notch shows a mountain shape (Widow’s peak); The fibula notch is relatively flat and like a plane (Flat); The fibula, relatively far between the posterior tubercle, will contact or be located very close from the anterior tubercle of the fibula notch, the gap is trapezoidal (Trapezoid); The fibular notch is concave and like a crescent (Crescent) (Fig. 3).

**Fig. 4:**
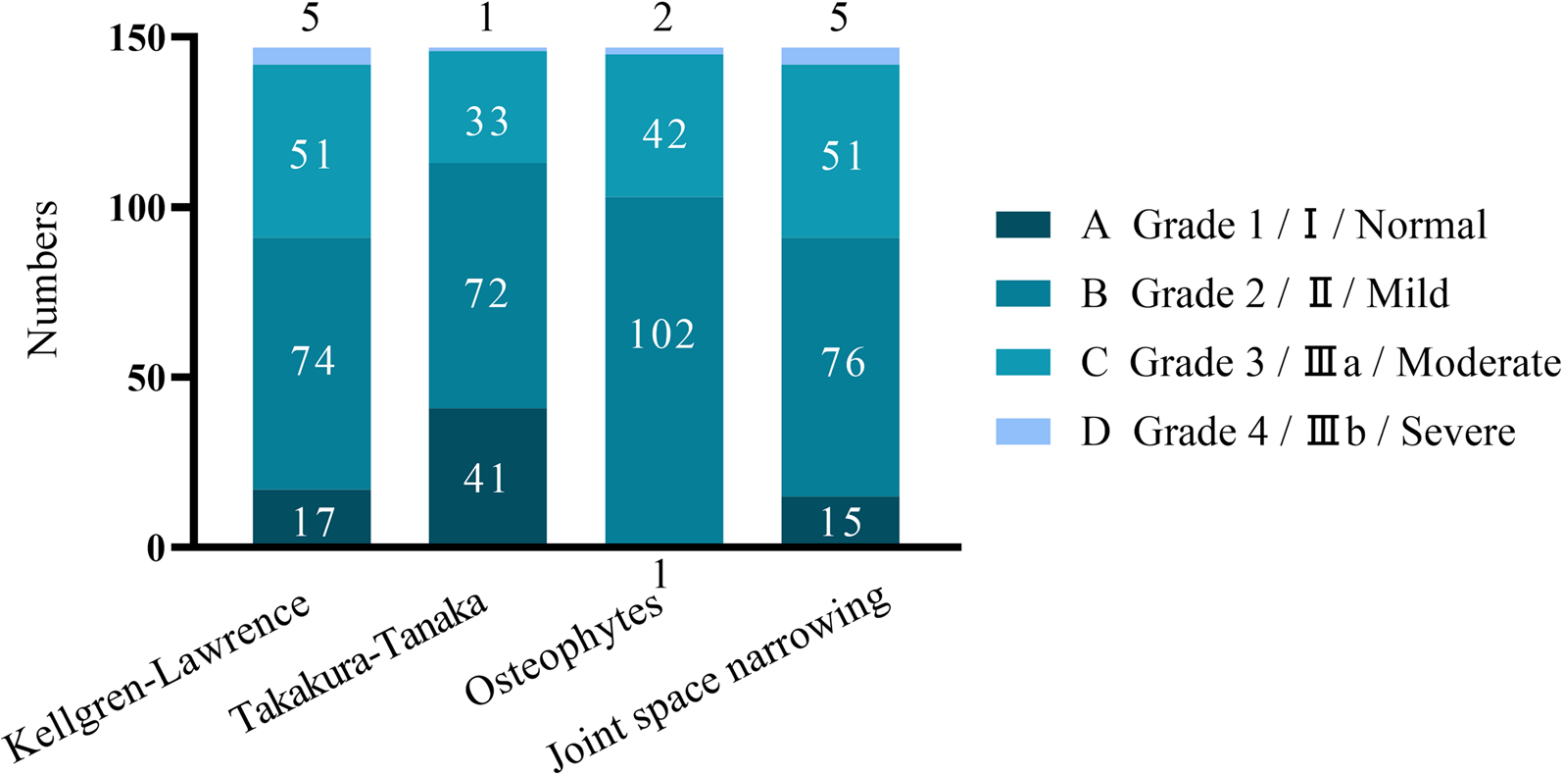
The proportion of patients in the modified Kellgren–Lawrence grade and the Takakura–Tanaka classification, osteophytes, joint space narrowing. The total number for each column is 147. In Kellgren–Lawrence, A: Grade 1, B: Grade 2, C: Grade 3, D: Grade 4. In Takakura–Tanaka, A: I, B: II, C: IIIa, D: IIIb. In osteophytes and joint space narrowing, A: Normal, B: Mild, C: Moderate, D: Severe

Table 1 reveals the parameters for MRI and radiological assessment. Interobserver reliability was good for all parameters on MRI (ICC = 0.676–0.899) and X-ray (ICC = 0.717–0.894) (*P* < 0.001). It shows the results of the tibiofibular distance, and the angle among the anterior and the posterior facets of the fibular notch are presented. The osteophytes, joint space narrowing, modified Kellgren–Lawrence grade, and the Takakura–Tanaka classification of all patients were shown (Fig. 4). It can be found that the patients are mainly concentrated in grade I and grade II for Takakura–Tanaka. The degree of osteophytes, joint space narrowing, and the modified Kellgren–Lawrence grade are primarily focused on grade 2 and grade 3.

**Table 1:** The parameters for MRI and radiological assessment

	Mean (SD)	Range		Mean (SD)	Range
a (cm)	0.48 (0.18)	0.20, 1.32	TAS	88.26 (3.20)	81.30, 102.90
b (cm)	0.86 (0.25)	0.16, 1.63	TTA	87.83 (7.47)	71.60, 99.60
c (cm)	0.41 (0.15)	0.12, 1.04	TT	1.55 (1.70)	0, 11.60
d (°)	138.80 (13.38)	100.30, 174.10	TLS	81.44 (3.54)	72.50, 89.50

a: The distance between anterior tibial tubercle (Chaput) and most anterior tubercle of the fibula; b: The distance between posterior tibial tubercle (Volkmann) and most posterior tubercle of the fibula; c: The maximum vertical distance between the medial fibular cortex and the anterior and posterior tubercle tips of the tibia; d: The angle between the anterior and posterior facets of the fibular notch of the tibia; *TAS* Tibial articular surface angle; *TTA* Talar tilt angle; *TT* Tibiotalar surface angle; *TLS* Tibial lateral surface angle

**Fig. 5:**
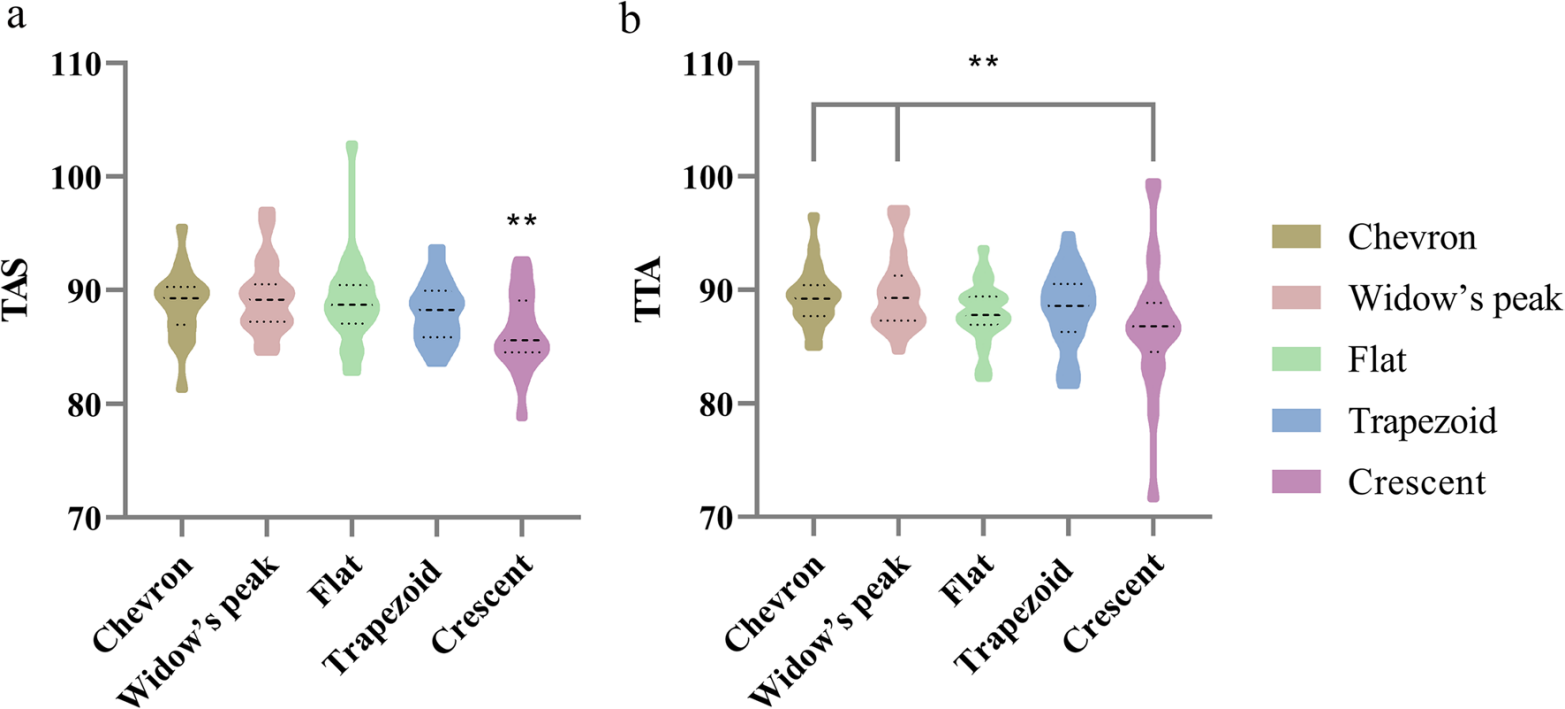
Differences between TAS and TTA in each classification. TAS: Tibial articular surface angle; TTA: Talar tilt angle (**P < 0.05)

Except for the TAS (*P* = 0.009) and TTA (*P* = 0.014), no statistical differences could be detected in the grade and other parameters among five classifications. The results are shown in Table 2. The TAS (degree) of the Crescent (86.47 ± 3.21) was less than Chevron (88.75 ± 2.72) (*P* = 0.006), Widow’s peak (89.26 ± 3.15) (*P* = 0.001), Flat (88.83 ± 3.62) (*P* = 0.003) and Trapezoid (88.11 ± 2.62) (*P* = 0.041), respectively (Fig. 5a). The TTA (degree) of Crescent (86.83 ± 5.30) was less than Chevron (89.28 ± 2.46) (*P* = 0.009) and Widow’s peak (89.82 ± 3.41) (*P* = 0.003) (Fig. 5b).

**Table 2:** Relationship between the DTS classification and ankle OA

	Angle				Osteophyte	Joint space narrowing	Grade	
	TAS	TTA	TT	TLS			Kellgren–Lawrence	Takakura–Tanaka
P value	**0.009**	**0.014**	0.731	0.809	0.056	0.224	0.180	0.509

*TAS* Tibial articular surface angle; *TTA* Talar tilt angle; *TT* Tibiotalar surface angle; *TLS* Tibial lateral surface angle; There were statistical differences in TAS (P = 0.009) and TTA (P = 0.014) among five classifications

**Fig. 6:**
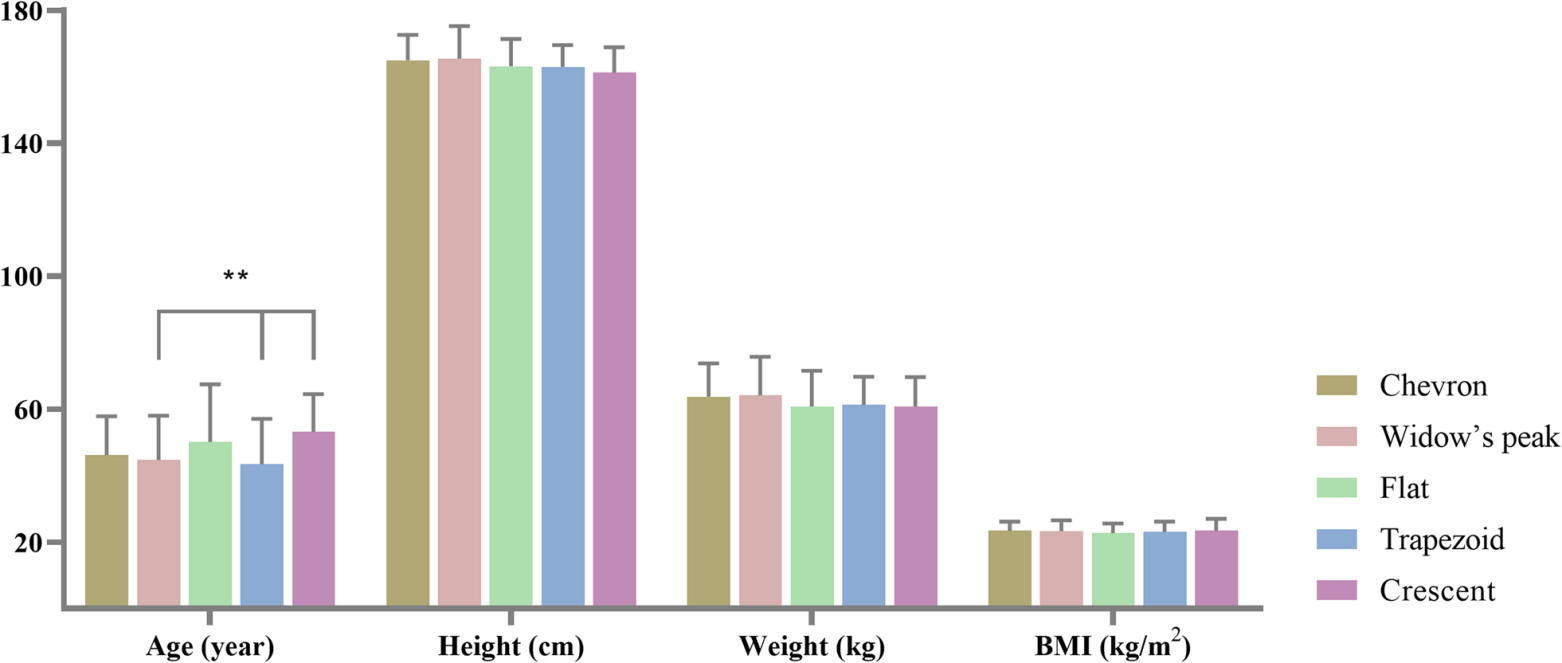
Baseline of participants based on DTS morphology classifications (**P < 0.05)

Moreover, there were significant differences in age among different classifications (*P* = 0.043) (Table 3). Patients of Crescent (53.2 ± 11.4) years are generally older than Trapezoid (43.5 ± 13.6) years (*P* = 0.007) and Widow’s peak (44.7 ± 13.4) years (*P* = 0.026). There were no statistical differences from others (Fig. 6). For the gender and left–right differences of ankle OA, the men were higher than women for the mean of TAS (*P* = 0.008) and angle (*P* = 0.003). Consistently, there were statistical differences among genders in osteophyte (*P* = 0.019) and the modified Kellgren–Lawrence grades (*P* = 0.041). But no statistical difference could be detected between the left and right foot’s angles with the numbers available (Table 4).

**Table 3:** Baseline of participants based on DTS classification (n = 147)

	Chevron (n = 29)	Widow’s peak (n = 24)	Flat (n = 33)	Trapezoid (n = 32)	Crescent (n = 29)	P value
Age (years)	46.20 (11.70)	44.70 (13.40)	50.20 (17.30)	43.50 (13.60)	53.20 (11.40)	**0.043**
Height (cm)	165.00 (7.60)	165.50 (9.80)	163.20 (8.20)	163.00 (6.60)	161.30 (7.60)	0.318
Weight (kg)	63.80(10.00)	64.20 (11.60)	60.80 (10.80)	61.30 (8.50)	60.80 (8.90)	0.534
BMI (kg/m^2^)	23.40 (2.80)	23.30 (3.20)	22.70 (2.90)	23.10 (3.10)	23.40 (3.60)	0.906

**Table 4:** Gender and left–right differences of ankle OA

	Men	Women	P value	Left	Right	P value
a (cm)	0.50(0.20)	0.45(0.17)	0.255	0.46(0.15)	0.50(0.20)	0.343
b (cm)	0.89(0.26)	0.81(0.23)	0.328	0.88(0.24)	0.84(0.26)	0.269
c (cm)	0.42(0.17)	0.40(0.13)	0.751	0.39(0.12)	0.44(0.18)	0.280
d (°)	140.55(14.34)	136.29(11.22)	**0.003**	137.35(11.93)	140.29(14.47)	0.311
TAS	88.87(3.30)	87.38(2.83)	**0.008**	88.02(3.12)	88.45(3.28)	0.711
TTA	88.91(3.55)	87.64(3.64)	0.074	88.40(3.84)	88.54(3.60)	0.744
TT	1.44(1.68)	1.71(1.74)	0.096	1.54(1.84)	1.56(1.57)	0.419
TLS	81.46(3.46)	81.40(3.68)	0.886	81.09(3.70)	81.80(3.36)	0.347
Osteophytes	–	**0.019**	–	0.221		
Space narrowing		0.055		0.504		
Kellgren–Lawrence		**0.041**		0.437		
Takakura–Tanaka		0.205		0.767		

a: The distance between anterior tibial tubercle (Chaput) and most anterior tubercle of the fibula; b: The distance between posterior tibial tubercle (Volkmann) and most posterior tubercle of the fibula; c: The maximum vertical distance between the medial fibular cortex and the anterior and posterior tubercle tips of the tibia; d: The angle between the anterior and posterior facets of the fibular notch of the tibia; *TAS* Tibial articular surface angle; *TTA* Talar tilt angle; *TT* Tibiotalar surface angle; *TLS* Tibial lateral surface angle; The men were higher than women for the angle (P = 0.003) and TAS (P = 0.008). There were statistical differences between genders in osteophyte (P = 0.019) and the modified Kellgren-Lawrence grades (P = 0.041)

## Discussion

We confirmed these five DTS classifications of ankle OA patients from cross-sectional MRI images. In statistics, although there was no link between DTS classification and severity of ankle OA, such as grade, the TTA, and TAS of Crescent tend to decrease compared with other classifications. In our study, the morphological characteristic of DTS is helpful for further understanding ankle OA.

Our results showed differences in TAS for various classifications. It can stop the distal fibula to forward translating if the anterior tubercle of the tibial is larger than the posterior tubercle [24]. Therefore, there is reason to speculate that the shape of the fibula notch affects the biomechanical structure and alignment of the tibia. Meanwhile, low TAS may again lead to recurrent instability and increased TTA for postoperative chronic ankle instability [25]. It is necessary to pay more attention to the changes in TAS and the development of ankle OA. According to the results, the Crescent were relatively older and had smaller TAS. With the progression of OA, the medial DTS space may become smaller or even disappear until terminal stages. The prognosis for patients undergoing surgical treatment at this time is likely to be poor. But the Crescent were not the most among included patients. Therefore, it should make timely interventions to prevent the inversion instability of the ankle after injury. Syndesmosis injuries caused ankle instability with medial traction force and external rotation torque to the tibia [26], which can explain the differences in TAS between different classifications in this study. Because of “subtalar compensation”, it appears that talar tilt can change the TAS [27]. Furthermore, DTS morphology structures will affect the subtalar joint, which may suggest “tibiofibular feedback” in ankle OA. In our known studies, the fibula will translate forward with aging, narrowing the distal syndesmosis [28]. This is consistent with our findings. Patients with Trapezoid younger than others have a relatively-far distance between the fibula and the posterior tubercle of the tibia. Moreover, because of the thinning of the junction cartilage and the change of joint space, the depth of the fibular notch will be affected by the increase in age. Patel et al. [29] have found that the fibular translation of men is more lateral than women. In this study, men are larger than women regarding the angle between the anterior and posterior parts of the fibular notch. And we further found the consistent result of the TAS.

We confirmed the five DTS morphological characteristics of 147 patients with ankle OA mainly based on the fibular notch and the position between tibia and fibula. Most of the patients included were PTOA, mainly secondary to ankle fractures or ligaments injury. The intrinsic stability of DTS is mainly dependent on the osseous structure of the fibular notch. In other words, we speculated that specific syndesmosis anatomical features are making the ankle joint more easily injured. The fibular incisure of Flat is shallowest and is almost straight. With the shallow depth of the fibular notch, it is more susceptive to increase the ability of anterior displacement of the fibula, leading to an increased risk of injury for the anterior talofibular ligament [15]. To further explore this issue, we compared the relevant parameters (a, b, c, d) between different classifications.

The results showed that d (degree) of the Flat was more than Chevron (129.93 ± 8.97) (*P* < 0.001), Crescent (130.38 ± 9.65) (*P* < 0.001), Trapezoid (139.17 ± 13.00) (*P* < 0.001), Widow’s peak (144.08 ± 12.29) (*P* = 0.049), respectively. The larger the d (degree), the shallower the fibular incision. Besides, a (0.54 ± 0.19), b (0.95 ± 0.31), and c (0.44 ± 0.17) of the Flat were all larger than the other four classifications. The shallow and separated syndesmosis is more prone to ligament damage [30]. Therefore, it may be one of the reasons why the proportion of the Flat in this study was the highest. The d (degree) of the Chevron is the smallest, and the fibular notch presents a clear angle. So, it is reasonable to speculate the risk of ligament damage is relatively small for the Chevron. Widow’s peak is similar to that of the Chevron and Crescent, but the d (degree) is larger compared to these classifications (*P* < 0.001). And the c of Widow’s peak (0.35 ± 0.14) is shallower than Flat (0.44 ± 0.17) (*P* = 0.035). It means that although the Widow’s peak is the least in the included patients, there is also the possibility of prior injury. Trapezoid can be seen as a further classification of the Flat. And the former (0.45 ± 0.17) has a smaller distance from the anterior tubercle of the tibia and fibula compared with the latter (0.54 ± 0.19) (*P* = 0.027). Therefore, the anterior tibial tubercle may be deficient in preventing the forward translation of the distal fibula. This may increase the risk of developing ankle instability after injury [11]. However, these hypotheses need to be determined in different comparative studies.

With the extensive use of CT scanning, the ligaments and bony features of DTS have attracted wide interest again recently. The weight-bearing CT (WBCT) imaging is an excellent and reliable way to evaluate the dynamic and biomechanical characteristics of syndesmosis [31, 32]. It has identified various specific morphologies that may affect the intrinsic or osseous stability of DTS based on WBCT and three-dimensional imaging (3D) analysis [33]. And 3D imaging from WBCT scans allowed for the complete quantification of syndesmotic injuries and provided more reliable information on ankle alignment compared with 2D measurements [34, 35]. Peiffer et al. [36, 37] have further developed a statistical shape model and discrete element analysis framework for CT images and explored syndesmotic ligament injury and abnormal mechanics of ankle OA. In the future, it is vitally necessary to understand the effects of DTS anatomical morphology on the ankle joint through dynamic 3D imaging, biomechanics studies, and gait analysis.

This study mainly found that various DTS morphology classifications are associated with TTA and TAS. It suggested that it is necessary to pay attention to the influence and appropriate management of the tibia and fibula for ankle OA. However, the relationship between different DTS morphology structures and past injuries remains inconclusive for PTOA. There are some limitations to the study. First, it did not guarantee that there will have no other new classifications because of the complex syndesmosis structure. We chose to confirm the general classification in this study. Second, the samples are restricted, and the trial size needs to be improved. However, no relevant clinical studies have been found for reference, so as many patients as possible were included based on the study site and period. Third, although there were some associations between the characteristics of the DTS and OA anatomy, the interaction biomechanical mechanism needed to be clarified. Further well-designed prospective controlled trials are required.

## Conclusions

The study results indicated that DTS classification might affect the biomechanics properties of TAS and TTA. TAS and TTA were relatively minor in ankle OA patients with DTS Crescent. Meanwhile, gender and age will affect DTS classification and ankle OA. These morphological characteristics are helpful for further study of the process of ankle OA. Further prospective controlled trials are needed to explain the relationship between syndesmosis structures and ankle OA severity in the future. A preprint has previously been published [38].

## Data Availability

A complete list of all included papers is available upon reasonable request from the corresponding author.

## Abbreviations

OA: Osteoarthritis
DTS: Distal tibiofibular syndesmosis
MRI: Magnetic resonance image
AITFL: Anteroinferior tibiofibular ligament
PITFL: Posteroinferior tibiofibular ligament
IOL: Interosseous ligament
ITL: Inferior transverse ligament
PTOA: Post-traumatic osteoarthritis
ICC: Intraclass correlation coefficient
SD: Standard deviation
TAS: Tibial ankle surface angle
TLS: Tibial lateral surface angle
TTA: Talar tilt angle
TT: Tibiotalar surface angle
WBCT: Weight-bearing CT
3D: Three-dimensional imaging
